# Preparation, characterization, and protective effects of *Gardenia fructus* carbon dots against oxidative damage induced by LPS in IPEC-J2 cells

**DOI:** 10.3389/fcimb.2024.1423760

**Published:** 2024-12-18

**Authors:** Bai-lu Chen, Xin-yi Zang, Jia-rong Mo, Ruo-yi Zhang, Heng Wang, Quan-xi Wang, Jian Li

**Affiliations:** ^1^ Fujian Key Laboratory of Traditional Chinese Veterinary Medicine and Animal Health, College of Animal Science, Fujian Agriculture and Forestry University, Fuzhou, China; ^2^ University Key Laboratory for Integrated Chinese Traditional and Western Veterinary Medicine and Animal Healthcare in Fujian Province, Fuzhou, China

**Keywords:** *Gardenia fructus*, carbon dots, IPEC-J2 cells, oxidative damage, characterization

## Abstract

This study aimed to prepare *Gardenia fructus* carbon dots (GF-CDs) and examine their efficacy in mitigating oxidative stress and apoptosis in intestinal porcine epithelial cells from the jejunum (IPEC-J2 cells) induced by lipopolysaccharide (LPS). The GF-CDs were synthesized using a one-step hydrothermal method. The oxidative damage model of IPEC-J2 cells was induced through LPS treatment. The potential mechanism by which GF-CDs affect cellular oxidative damage was examined through the perspectives of apoptosis, reactive oxygen species level, antioxidant-related enzyme index, mRNA transcription of antioxidant-related genes, and the expression of antioxidant proteins. The results revealed that GF-CDs, characterized by particle sizes<7 nm, abundant functional groups, and good water solubility, were synthesized using a one-step hydrothermal method. The carbon spots of *Gardenia fructus* at concentrations of 50, 100, and 200 μg/mL exhibited protective effects, as evidenced by their ability to enhance viability (*P*<0.01) and restore cellular morphology after oxidative damage. The GF-CDs decreased oxidative damage and reduced the apoptosis rate of cells by upregulating AKT1 expression and downregulating the expression of Caspase 3, STAT3, TNF-α, and JNK. These results indicate that GF-CDs have the characteristic physicochemical properties of CDs, exhibit biological activities related to antioxidation and cellular damage mitigation, and may serve as a potential healthcare product in swine raising.

## 1 Introduction

The basis for swine farming for maintaining homeostasis is the proper raising of pigs, as oxidative stress frequently triggers various diseases that disrupt the balanced development of swine raising and impact economic efficiency. Swines experiencing oxidative stress are prone to decreased feed efficiency, reduced appetite, and stunted growth. Additionally, piglets frequently exhibit impaired growth and increased mortality rates, which can significantly affect the overall production efficiency and meat quality of the herd (Bacou et al., 2021). Prolonged stress diminishes the antioxidant capacity of the swine herd, resulting in immune system dysregulation. Coupled with inadequate nutrient absorption, this leads to a significant decrease in disease resistance, increasing susceptibility to illness and death (Hong et al., 2024). As the primary organ for nutrient digestion and absorption in swine, the intestine is an integral part of the immune system and is susceptible to intestinal diseases after oxidative stress.

Many studies reported that incorporating Chinese herbs or their active ingredients into feed can significantly prevent oxidative damage and restore physiological functions. Chinese herbal medicines are used in clinical practice because of their minimal side effects and absence of drug residues, with many exhibiting antioxidant properties suitable for practical application.

The antioxidant properties of *Gardenia jasminoides* Ellis (GF) have been extensively validated (Tian et al., 2022; Zhang X. et al., 2023), and its distribution and cultivation are extensive, with abundant resources (Jin et al., 2023). GF is a popular shrub from the Rubiaceae family, scientifically called Yellow Gardenia Jasminoides Ellis and Mountain Gardenia Jasminoides Ellis. The dry and ripe fruits of this herb are commonly used for medicinal purposes. *Gardenia* exhibits medicinal properties that are bitter and cold, affecting the heart, lungs, and sanjiao meridian. The active ingredients in *Gardenia* (genocide and crocin) can scavenge free radicals and exert antioxidant effects (Jiang et al., 2021). In 2002, the former National Ministry of Health officially recognized GF as a dual-use traditional Chinese medicine and was included in the inaugural batch of the medicine-food homology list. Due to its low toxicity and extensive pharmacological effects, GF has been utilized as feed additives or clinical prescription drugs in livestock and poultry to enhance disease resistance and diminish antibiotic usage (Chen et al., 2020).

**Figure 1:**
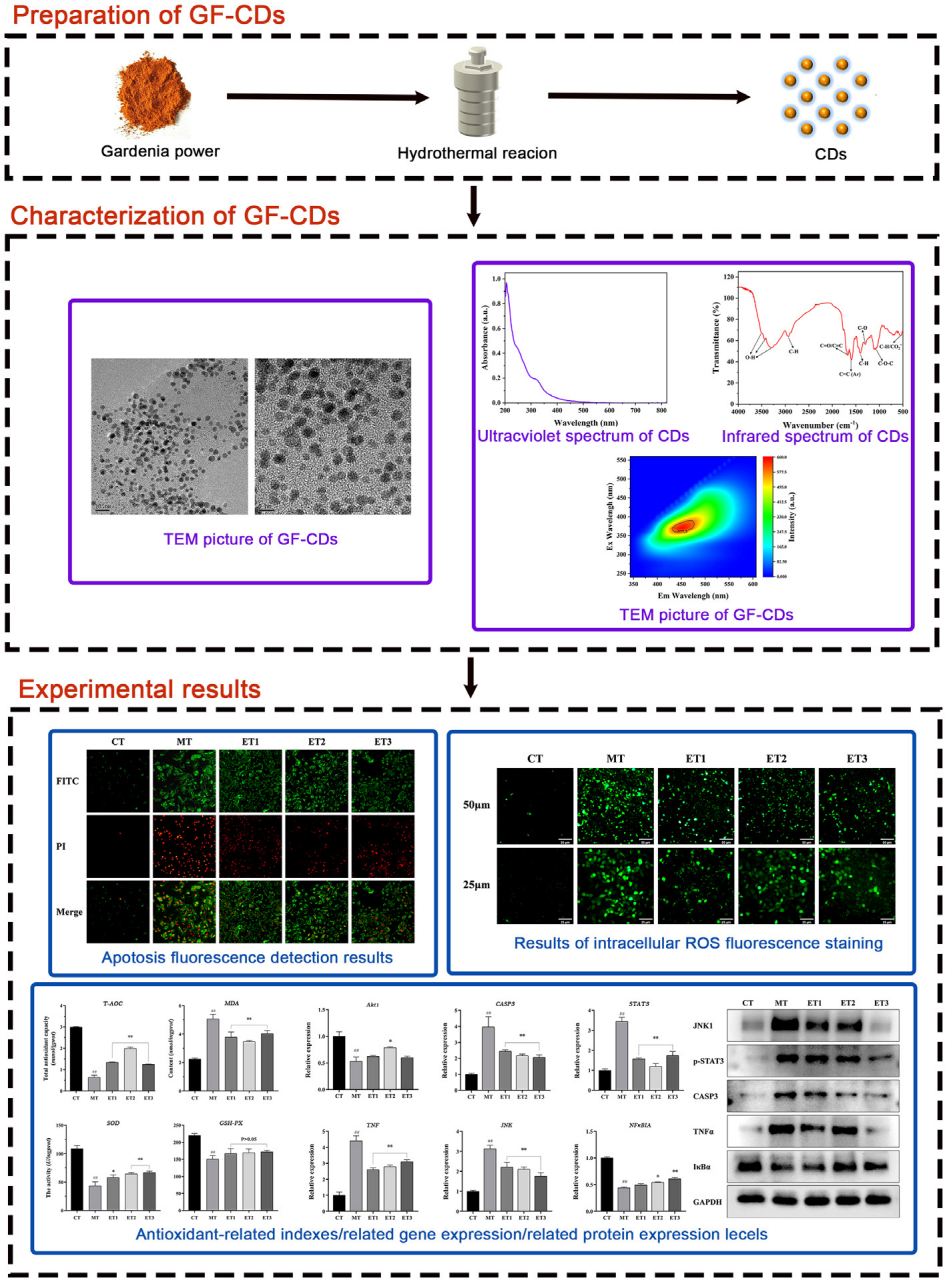
Flow chart of the experimental approach.

Although GF exhibits significant antioxidant properties, its bitter taste may adversely affect pig feed consumption. Therefore, modifying the formulation to reduce the required dosage is imperative. Carbon dots (CDs), referred to as carbon quantum dots or carbon nanodots, are fluorescent nanoparticles characterized by a particle size distribution<20 nm (Wei et al., 2023). CDs have significant research potential. The new nanometer traditional Chinese medicine dosage form preserves the original efficacy of traditional Chinese medicine significantly. Moreover, it has several characteristics, including low dosage, high water solubility, and good biocompatibility (Lu and Li, 2018; Li and Zhuang, 2019). Therefore, we hypothesized that the integration of *Gardenia* in swine farming can be enhanced through CDs formulation. Furthermore, we aimed to examine whether the CDs obtained from Gardenia maintain substantial biological activities, including antioxidant properties and cellular protection. Based on related research, we successfully synthesized GF-CDs as a precursor and accomplished micro-addition while preserving the therapeutic properties of the *Gardenia.* No reports exist on the synthesis of CDs from Chinese herbs, no relevant examples of CDs synthesized from *Gardenia*, and no studies have investigated the mechanism of antioxidant damage of *Gardenia* from the perspective of nano-like components. Therefore, this study aimed to examine the structural characteristics of GF-CDs and their intervention on the intestinal porcine epithelial cell line (IPEC-J2 cells), a standard model for studying intestinal oxidative stress in swine (Li et al., 2024). **Figure 1** depicts the process of this study.

## 2 Materials and methods

### 2.1 Materials, chemicals, and reagents

GF was obtained from the Fujian Province of China. Dr. Li Jian of Fujian Agriculture and Forestry University (FAFU) morphologically authenticated the fruiting body of GF, and the corresponding voucher specimens were deposited in Fujian Key Laboratory of Traditional Chinese Veterinary Medicine and Animal Health of FAFU (Voucher number NO. FAFU-TCVM-20210017). The herb is produced in Quanzhou, Fujian Province. IPEC-J2 cells were procured from the ATCC of the United States (Li et al., 2024). This study utilized exclusively cells from passages 3 to 6. The cells used in each group for each experiment were of identical generation.

The cell counting plate was obtained from Watson, Japan. The Dulbecco’s Modified Eagle Medium (DMEM), superior fetal bovine serum, penicillin/streptomycin double antibody solution, and 0.25% Trypsin-EDTA (1×) were all procured from Gibco. The 96-well culture plates and T25 cell culture bottles were obtained from Wuxi Nesi Biotechnology Co., Ltd. In addition, the 0.22 μm microporous membrane and ready-to-use dialysis bag used in the experiment were obtained from Millipore and Beijing Solibao Technology Co., Ltd. The Milli-Q Direct 8/16 ultrapure water system procured from Merck Millipore (USA) was used in all the experiments.

### 2.2 Preparation of GF-CDs

The *Gardenia* was washed, dried at low temperature, crushed, and placed in a sealed bag to protect it from light. We measured 4 g of *Gardenia* powder, added 65 mL of deionized water, and stirred them for 30 min until thoroughly mixed. Subsequently, the mixture was transferred into a 100 mL reaction kettle, placed in the oven, set to 200°C, and reacted for 7 h (Gholipour and Rahmani, 2024). After natural cooling, we centrifuged the reaction solution at 9,500 rpm for 25 min to remove the residue of *Gardenia* powder agglomerated large particles and filtered the supernatant through a 0.22 μm filter membrane. A dialysis bag (MW: 500–1,000 Da) was used to dialyze the reaction solution in ultrapure water for 24 h, during which the ultrapure water was changed every 2 h (Zhang Y. et al., 2023). The product was concentrated, frozen for 10 h, freeze-dried, and acquired as a dark brown product, which was stored in a dry environment away from light and reserved (**Figure 1**).

### 2.3 Characterization of GF-CDs

Transmission electron microscopy (TME) (TECNAI G2 20, FEI Company, USA) was used to examine the solution’s size, morphological characteristics, and distribution of GF-CDs. A trace UV spectrophotometer (Nanodrop 2000c) (Thermo) was used to detect the UV-absorbable light visible spectrum (UV-vis). The UV-vis detection function of the instrument was selected, and the spectrogram data were obtained using the wavelength range set as 200–800 nm (Gholipour and Rahmani, 2024).

A Fourier infrared spectrum analyzer (Bruker, Germany) was used to detect the infrared spectrum (IR) of the GF*-*CDs. A fluorescence spectrometer (Cary Eclipse) (Agilent) was used to obtain the fluorescence spectrum (PI) of the GF-CDs; the excitation slit and emission slit were set to 10 nm, whereas the scanning speed was set to 1,200 nm/min. The emission spectrum was scanned at varying excitation wavelengths, with a test wavelength range of 250–750 nm (Hu et al., 2021; Zhao et al., 2020).

### 2.4 Determination of cell viability and establishment of the oxidative damage model

#### 2.4.1 Toxicity test of GF-CDs on IPEC-J2 cells

The IPEC-J2 cell suspension concentration was calibrated to 7×10^4^ cells/mL, inoculated into 96-well plates at 100 μL per well, and cultured until the cells adhered, after which the supernatant was aspirated and discarded. Subsequently, the corresponding medium, carbon point solution, or LPS solution was added according to the experimental grouping and executed as follows:

We added only DMEM medium to the control group, and the experimental groups were treated with 50, 100, 200, 400, 600, and 800 mg/mL of GF-CDs solution, respectively, acting at 100 mL per well for 24 h to determine the biotoxicity of GF-CDs on IPEC-J2 cells. The experiment was divided into seven groups; six replicate wells were set up for each group, and the experiment was repeated thrice.

A model of oxidative cell damage was established by preparing 1 mg/mL LPS solution, which was subsequently added to the DMEM medium and diluted to various concentrations for experimentation. In the control group, only DMEM medium was administered, while in the experimental group, based on the pre-test findings and references (Yu et al., 2022; Han et al., 2024), LPS solutions of 2.5, 5, 10, 20, 40, 50, 100, 200, and 400 μg/mL were added in equal volumes for 12 h. The experiment consisted of 10 groups, each containing six replicate wells, and was repeated thrice.

After the above operation, 20 μL of MTT solution (5 mg/mL) was added to each well and incubated for an additional 4 h. The supernatant was discarded, 150 μL of DMSO was introduced and shaken, and the absorbance (OD) of each well at 490 nm was measured using an enzyme marker. Cell viability was calculated according to the following equation.

#### 2.4.2 Screening of concentration of the GF-CDs against oxidative damage

Cell viability was assessed using an MTT assay, and the optimal concentration of GF-CDs was selected for subsequent analyses. Cells in the logarithmic growth phase were collected, and their concentration was calibrated to 7×10^4^ cells/mL. Subsequently, 100 μL of cells was seeded into a 96-well plate. DMEM medium was administered to the blank and the model groups. Furthermore, equal volumes of GF-CDs solution (50, 100, 200, 400, 600, and 800 μg/mL) were administered to the other groups for 24 h. Subsequently, the supernatant was discarded, and the DMEM medium was introduced to the blank group. The model and GF-CDs concentration groups were incubated with the specified concentration of LPS for 12 h. Subsequently, 20 μL of MTT solution was added to each well, and the culture was kept in the dark for 4 h. The supernatant was discarded, and 150 μL of DMSO was introduced and shaken. Cell viability was calculated using a microplate reader. The experiment was repeated thrice, with each group containing six duplicates.

### 2.5 Experimental grouping

The results of the GF-CDs antioxidant damage prompted the division of subsequent tests into five groups: control group (DMEM medium only), model group (DMEM culture for 24 h followed by addition of 2.5 μg/mL LPS for 12 h), experimental group 1 (cultured with 50 μg/mL carbon for 24 h and treated with 2.5 μg/mL LPS for 12 h), experimental group 2 (100 μg/mL of GF-CDs culture for 24 h followed by addition of 2.5 μg/mL LPS for 12 h), and experimental group 3 (200 μg/mL of GF-CDs culture for 24 h followed by 2.5 μg/mL LPS for 12 h). They were abbreviated CT, MT, ET1, ET2, and ET3.

### 2.6 Observation and detection of intracellular reactive oxygen species (ROS) level

The levels of ROS within the cells were observed using the fluorescent probe DCFH-DA under an inverted fluorescence microscope. In addition, the fluorescence intensity was measured using flow cytometry. Before the experiment, DCFH-DA was diluted with DMEM medium at a ratio of 1:1000 to achieve a final concentration of 10 μM. After loading, it was stored at -20°C.

ROS levels were assessed using fluorescent staining. The IPEC-J2 cells were harvested during the logarithmic growth stage. The cells were seeded in 24-well plates and cultured until adherence was achieved. Subsequently, the IPEC-J2 cells were treated based on experimental groups. They were rinsed twice with PBS, diluted DCFH-DA was introduced, and incubated at 37°C for 25 min in the dark. The supernatant was washed thrice with PBS. An adequate volume of PBS was added, subsequently followed by immediate observation. The samples were photographed using an inverted fluorescence microscope.

The fluorescence intensity was analyzed using flow cytometry (Sözer et al., 2024). Cells in the logarithmic growth phase were harvested and seeded into six-well plates for culture. Subsequently, the cells were harvested through trypsin-EDTA digestion, centrifuged at 1,000 rpm for 5 min, and the supernatant was discarded. The cells were washed with DMEM once, resuspended in diluted DCFH-DA, and incubated at 37°C for 25 min in the dark, during which they were inverted every 5 min. The cells were washed with DMEM thrice and resuspended in PBS. Subsequently, the cells were detected using flow cytometry, and the experiment was repeated thrice.

### 2.7 Detection of antioxidant-related indicators

According to the experimental groups, IPEC-J2 cells in the logarithmic growth phase were seeded into six-well plates at 7×10^4^ cells/mL, and appropriate reagents were subsequently added for utilization. Upon completion of the treatment, the culture medium was aspirated and discarded. The cells were directly scraped off using a cell scraper. An appropriate volume of PBS was added to the cells before transferring them to the centrifuge tube. The cells were centrifuged at 1,000 rpm for 8 min, after which the supernatant was discarded. A specific volume of PBS buffer was added to the cell precipitate, and the cells underwent ultrasonication at 300 W in an ice-water bath. Each ultrasound was performed for 5 s with five repetitions. The ultrasound was performed at 30 s intervals. The acquired homogenate was utilized for subsequent analyses.

First, the protein concentration in cell homogenates across each experimental group was assessed using a bicinchoninic acid (BCA) protein detection reagent. This procedure was performed using the kit method. The total antioxidant capacity (T-AOC), superoxide dismutase (SOD) activity, glutathione peroxidase (GSH-PX) activities, and malondialdehyde (MDA) content of cell samples in each group were assessed using various detection kits according to the manufacturer’s instructions (Li et al., 2024). The experiment was repeated thrice.

The mRNA transcription of antioxidant-related genes was measured using quantitative polymerase chain reaction (qPCR). Cells in each experimental group were cultured in the 12-well plates with three replicates in each group. After the corresponding treatment, total RNA was extracted from the cells using an RNA extraction kit following the manufacturer’s instructions. The concentration and purity of the extracted RNA were analyzed using a microspectrophotometer. An RNA-protein ratio (A_260_/A_280_) between 1.8 and 2.0 indicates good purity, allowing the samples to be used for further analyses. The isolated RNA was reverse transcribed to cDNA utilizing a reverse transcription kit following the manufacturer’s instructions. The reverse transcription program involved incubation at 42°C for 15 min and reheating at 85°C for 5 min. The resultant cDNA was stored at -20°C. Based on the results of the pharmacologic network analysis of GF in our laboratory, potential gene targets for GF-CDs were identified. The sequence data of these genes was retrieved in the NCBI database. Primers were designed in accordance with the standard principles of primer design, and their accuracy was validated using primer-BLAST.

Subsequently, quantitative real-time PCR (qPCR) was performed. DEC-DDH2O was used to dilute the cDNA, and the SYBR Green I fluorescent dye method was used to perform the qPCR reaction. Each group of samples was set up in four replicates. **Table 1** presents the reaction system. The above reaction solution was added to the 384-well PCR plate, sealed with a membrane, and centrifuged at 2,400 rpm for 1 min at 4°C. **Table 2** presents the reaction conditions. GAPDH was used as the internal reference. The relative expression of each gene was calculated according to the following formula:

**Table 1:** Reaction system of qPCR.

Component	Volume(μL)
cDNA template	0.6
Forward primer(10μM)	0.2
Reverse primer(10μM)	0.2
2×RealStar Geen Fast Mixture	5
RNase-free H_2_O	Supplement until 10 μL

**Table 2:** Reaction condition of qPCR.

Step	Temperature (°C)	Duration	Number of cycles
Pre-denaturation	95	2min	1
Denaturation	95	15s	40
Annealing/Extension	60	20s	40

### 2.8 Assessment of the level of antioxidant-related proteins using Western blot

The cells were seeded in six-well plates, with each group comprising three replicates. The culture medium was administered based on the experimental group, and the protein was extracted after the treatment. The culture medium was discarded, and the sample was washed twice with pre-cooled PBS. Then, the cells were harvested using a cell scraper. A total of 150 μL of 1× RIPA lysate was added. Next, the lysate was blown, mixed, and lysed for 20 min on ice. The lysate was centrifuged at 12,000 rpm for 10 min at 4°C, and the supernatant was discarded. The protein concentration was assessed using a BCA assay reagent. An appropriate quantity of protein extract was measured, and 1/4 volume of 5× loading buffer was added. The mixture was thoroughly mixed, heated at 96°C for 10 min, and rapidly cooled on ice.

The electrophoresis tank was filled with prefab glue with a concentration of 4%–15%, followed by adding the electrophoresis solution. Subsequently, the ulnar comb was extracted. Each well contained 20 μg of protein sample for analysis, and 5 μL of pre-stained protein marker was added to both sides of the sample. The constant pressure of concentrated glue electrophoresis was set to 80 V for 30 min, and the pressure of separation glue electrophoresis was 120 V. The electrophoresis was concluded when bromophenol blue reached the bottom of the glue. The filter paper and the activated PVDF membrane were prepared and immersed in the membrane transmembrane solution for subsequent use. The gel was extracted from the glass plate, appropriately trimmed, and placed in the membrane transfer liquid. We spread filter paper, PVDF film, gel, and filter paper on the sandwich plate, removed the bubbles, and installed the printing clip. The power source of the film-rotating instrument was switched on, and the rotating process of the film was activated. A TBST-blocking buffer comprising 5% skim milk powder was formulated. After the membrane rotation, the filter paper was extracted, and the PVDF membrane was transferred to the blocking solution using forceps and gently shaken for 90 min using a shaker. The antibodies were incubated according to the instructions.

The ECL luminescent solution was added to the plastic wrap. The PVDF film was placed onto the plastic wrap, ensuring complete contact of the protein surface of the membrane with the solution. This was allowed to react for 1 min. The film was transferred to another plastic wrap, the residual liquid was removed, and the film was wrapped for tablet pressing. The exposure duration was modified according to the signal intensity, and the film was extracted after exposure. It was promptly immersed in the developing solution for 1 min and in the fixing solution for 8 min. All residual fixing liquid on the film was removed, and the film was air-dried before scanning. Image J software was used to analyze the gray value (Li et al., 2024).

### 2.9 Detection of apoptosis

Apoptosis of IPEC-J2 cells was examined using combined fluorescence microscopy and flow cytometry with FITC-V (Annexin V-FITC) and propidium iodide (PI) probes. Cell apoptosis was identified and analyzed using fluorescent staining. Cells in the logarithmic growth phase were harvested, inoculated into 24-well plates, cultured until adherent, and treated according to their respective experimental groups. The whole plate was centrifuged at 1,000 rpm for 3 min to collect cells that were shed due to apoptosis. PBS was extracted through spiration. Subsequently, 100 μL of 1× binding buffer, 5 μL of Annexin V-FITC, and 10 μL of PI staining solution were mixed gently into the wells. The samples were incubated in the dark at room temperature for 15 min and examined immediately using an inverted fluorescence microscope.

Apoptosis was identified using flow cytometry (Xu et al., 2021). Cells in the logarithmic growth phase were harvested and seeded into the six-well plates. The cells were treated based on experimental groups. Additionally, one control group was cultured without staining, and two model groups were created, one with Annexin V-FITC and the other with PI staining, for parameter adjustment. The cell culture medium was aspirated into a centrifuge tube, and the cells on the plate were washed with PBS. The cells were harvested using trypsin digestion without EDTA and transferred to a centrifuge tube containing the culture medium. The cells were centrifuged at 1,000 rpm for 5 min at 4°C. The cells were washed with pre-cooled PBS once, and the supernatant was discarded. Subsequently, 100 μL 1× binding buffer was introduced into the centrifuge tube for re-suspension, followed by adding 5 μL of Annexin V-FITC and 10 μL of PI Solution, which were gently mixed. After incubation at room temperature for 15 min in the dark, 500 μL of buffer was added, mixed, and placed on ice. Flow cytometry was used, and the test was repeated thrice.

## 3 Results

### 3.1 Characterization of the GF-CDs

**Figure 2:**
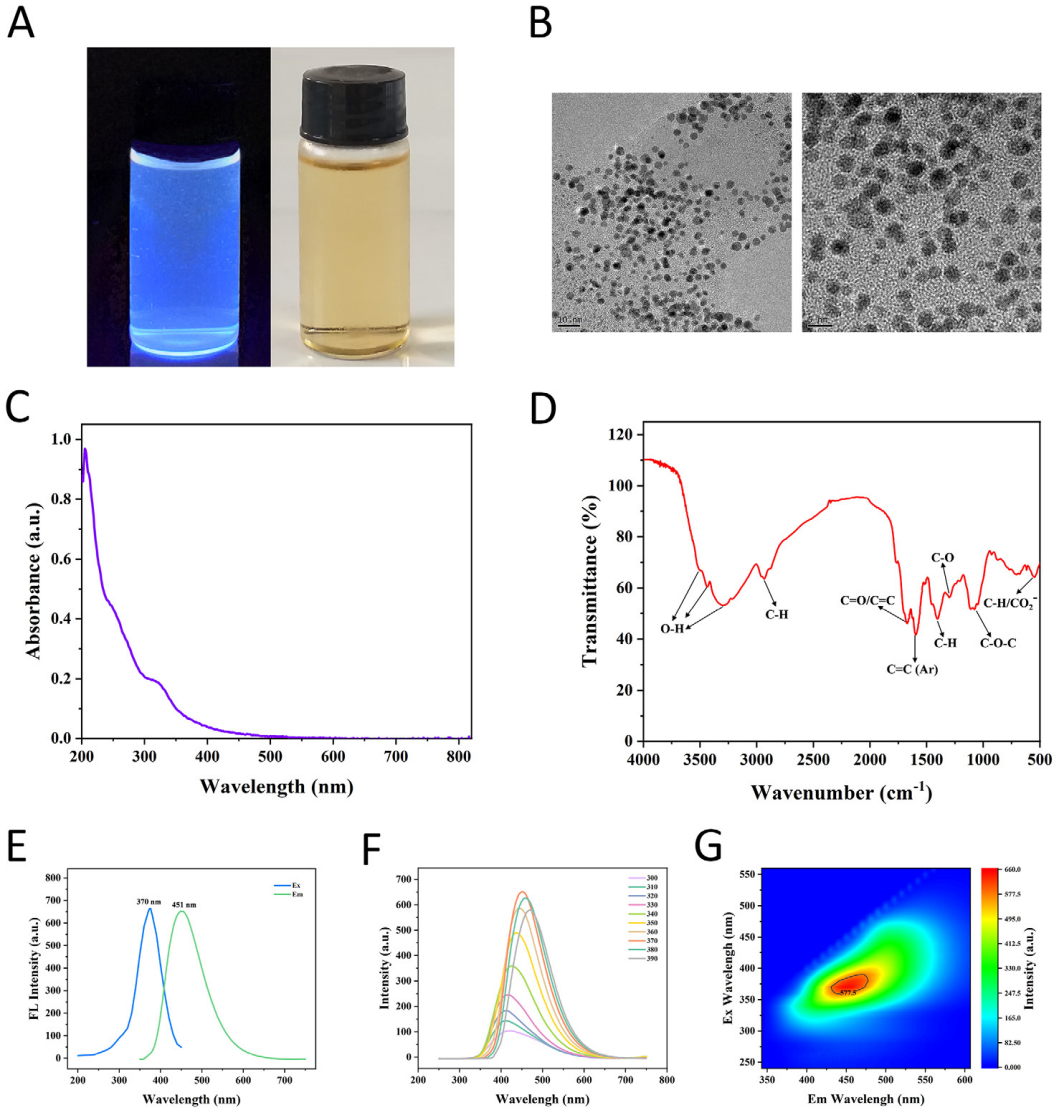
Morphological analysis and characterization of GF-CDs. **(A)** Color of GF-CDs solution in different light; **(B)** TEM pictures of GF-CDs; **(C)** Ultraviolet-visible absorption spectrum of GF-CDs; **(D)** Infrared spectrum of GF-CDs; **(E)** The best excitation spectrum; **(F)** Fluorescence emission spectra of different excitation wavelengths; **(G)** 3D fluorescence spectra.

The GF-CDs prepared using the hydrothermal method exhibited a clear yellowish color under natural light (right side of **Figure 2A**). Moreover, the GF-CDs emitted blue fluorescence under 365 nm ultraviolet light (left side of **Figure 2A**).

The TEM image (**Figure 2B**) illustrates that GF-CDs were uniformly distributed and well-dispersed within the 10 and 5 nm field of view. The GF-CDs were identified as spherical particles with particle sizes<10 nm. Statistical analyses revealed that the particle size of GF-CDs predominantly ranged from 2 to 7 nm, with a mean particle size of approximately 4.8 ± 0.52 nm.

The absorption spectrum of GF-CDs in the UV-visible light range is illustrated in (**Figure 2C**). The tail of the spectral line extends into the visible light spectrum. A weak UV absorption, as observed at a wavelength of 252 nm, is potentially attributed to the n-π* transition of the C=O bond. A shoulder peak at 312 nm is possibly attributed to the π-π* transition of the C=C bond.

The infrared spectrum of GF-CDs (**Figure 2D**) demonstrates multiple absorption peaks, signifying the abundance of functional groups on the surface of GF-CDs. The spectrogram analysis revealed that the broad absorption peak at 3,300–3,500 cm^–1^ is attributed to the O–H extension vibration. The absorption peak at 2,933 cm^–1^ was caused by C–H extension. The absorption peak at 1,671 cm^–1^ could be attributed to either C=O or C=C stretching. The absorption peak at 1,596 cm^–1^ is attributed to C=C stretching vibration in aromatic compounds. The absorption peak at 1,405 cm^–1^ results from C-H bending vibration. The peaks at 1,299 and 1,110 cm^–1^ result from C–O stretching and C–O stretching vibration in the ether group, respectively. Consequently, GF-CDs have hydrophilic groups, including hydroxyl and carbonyl, and may contain aromatic structures.

The optimal excitation spectrum of GF-CDs (**Figure 2E**) indicates an optimal excitation wavelength λ_Ex_ = 370 nm and a maximum emission wavelength λEm=451 nm, with the highest fluorescence emission intensity. The fluorescence emission spectrum of GF-CDs was analyzed at various excitation wavelengths (**Figure 2F**). It was found that (1) the fluorescence emission spectrum of GF-CDs is affected by the excitation wavelength, as the fluorescence emission peak progressively shifted toward longer wavelengths with increasing excitation wavelength; (2) the fluorescence intensity initially increased and subsequently decreased with an increasing excitation wavelength; (3) the fluorescence intensity reached its maximum at an excitation wavelength of 370 nm. The 3D fluorescence map of GF-CDs (**Figure 2G**) indicated that the fluorescence intensity was highest in the red region, with optimal excitation and emission wavelengths of approximately 370 and 450 nm, respectively, consistent with the results of the front row map.

### 3.2 Cellular toxicity of GF-CDs

**Figure 3:**
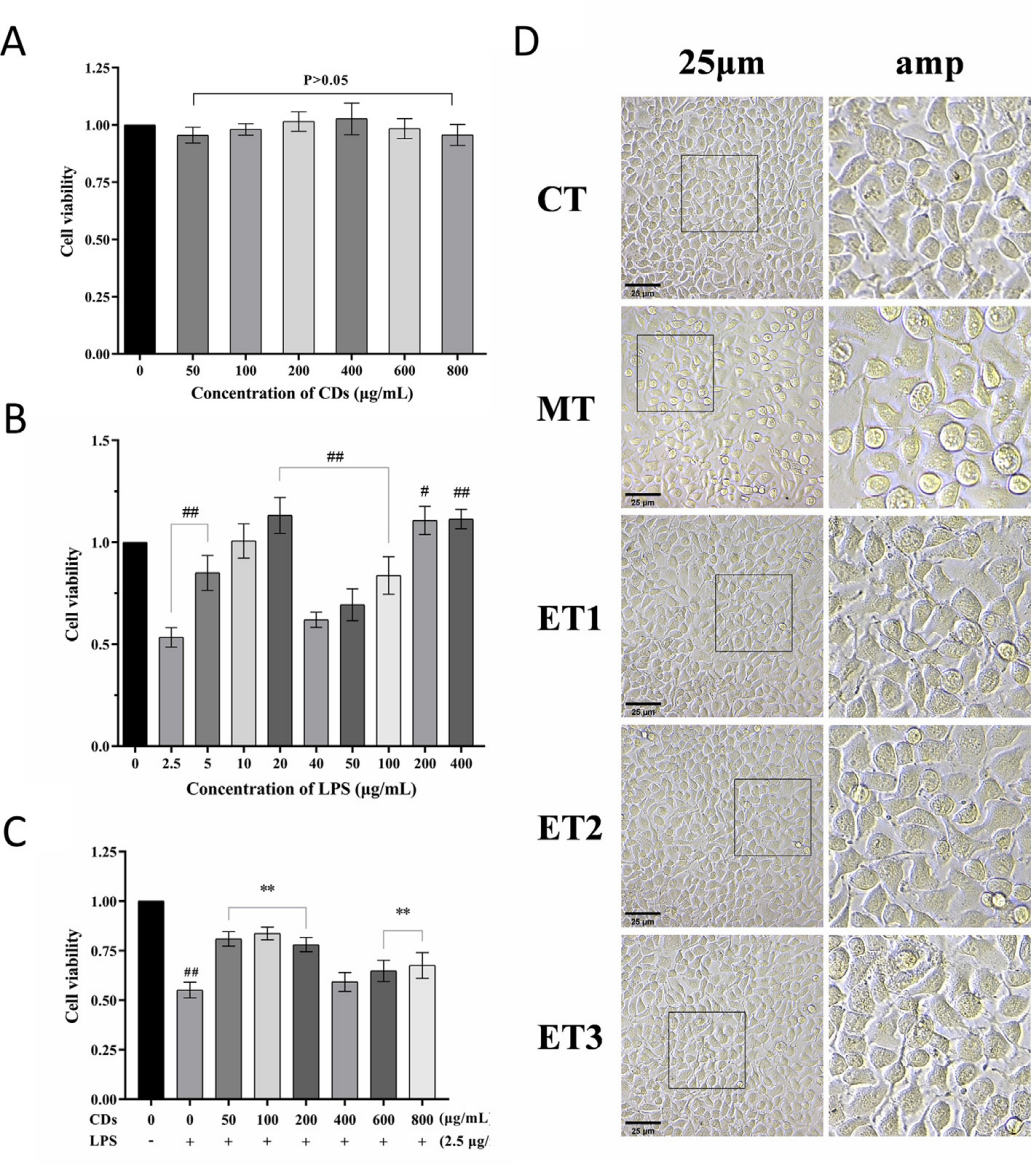
Effect of GF-s on the viability and morphology of oxidative damaged cells. **(A)** Cytotoxicity of GF-CDs; **(B)** Screening of the concentration of LPS induced oxidative damage; **(C)** Screening of antioxidant damage concentration of GF-CDs; **(D)** Observation of cell morphology. All values were presented as means ± SD, and the significance of the difference were represented by asterisks and pound signs. CT, control group; MT, model group; ET1, experimental group 1; ET2, experimental group 2; ET3, experimental group 3. Compared with the control group, ^#^ *P*<0.05, ^##^ *P*<0.01; Compared with the model group, *P<0.05, **P<0.01.

The MTT assay results are presented in (**Figure 3A**), indicating that the viability of IPEC-J2 cells across the six experimental groups remained at approximately 100% after 24 h of GF-CDs solution treatment, exhibiting no significant variation from the control group (*P*>0.05). This suggests that the GF-CD solution exhibited low toxicity to IPEC-J2 cells within this concentration range.

### 3.3 Effects of GF-CDs on the viability and morphology of oxidation-damaged cells

**Figure 3B** illustrates that varying concentrations of LPS elicited different changes in the viability of IPEC-J2 cells. All treatment groups exhibited significant differences from the control group except for the 10 μg/mL group (*P*<0.05). At a concentration of 2.5 μg/mL, cell viability was 53.41%, closely approximating half the viability observed in the control group. This concentration was considered optimal for LPS modeling. Following the introduction of GF-CDs, the cell viability in each GF-CDs concentration group was lower than that of the blank group but higher than that of the model group. Compared with the model group, 50, 100, and 200 μg/mL concentrations exhibited significantly higher cell viability (*P*<0.01) and exhibited the largest increase, indicating that these three concentrations exhibited the strongest intervention effect on IPEC-J2 cell injury, and they were selected as the treatment concentrations for subsequent analysis (**Figure 3C**).

Furthermore, the control group cells exhibited similar morphology with flat polygonal adherent growth, a clear structure, and a prominent nucleus. In the model group, cellular connections were tenuous; most cells were spheroid, with incomplete adherence, and few exfoliated cells were observed. Compared with the model group, the cells of the three experimental groups were closely connected and morphologically indistinguishable from those of the control group. Only a few cells were not completely adherent to the wall (**Figure 3D**).

### 3.4 Effects of GF-CDs on ROS levels in oxidation-damaged cells

**Figure 4:**
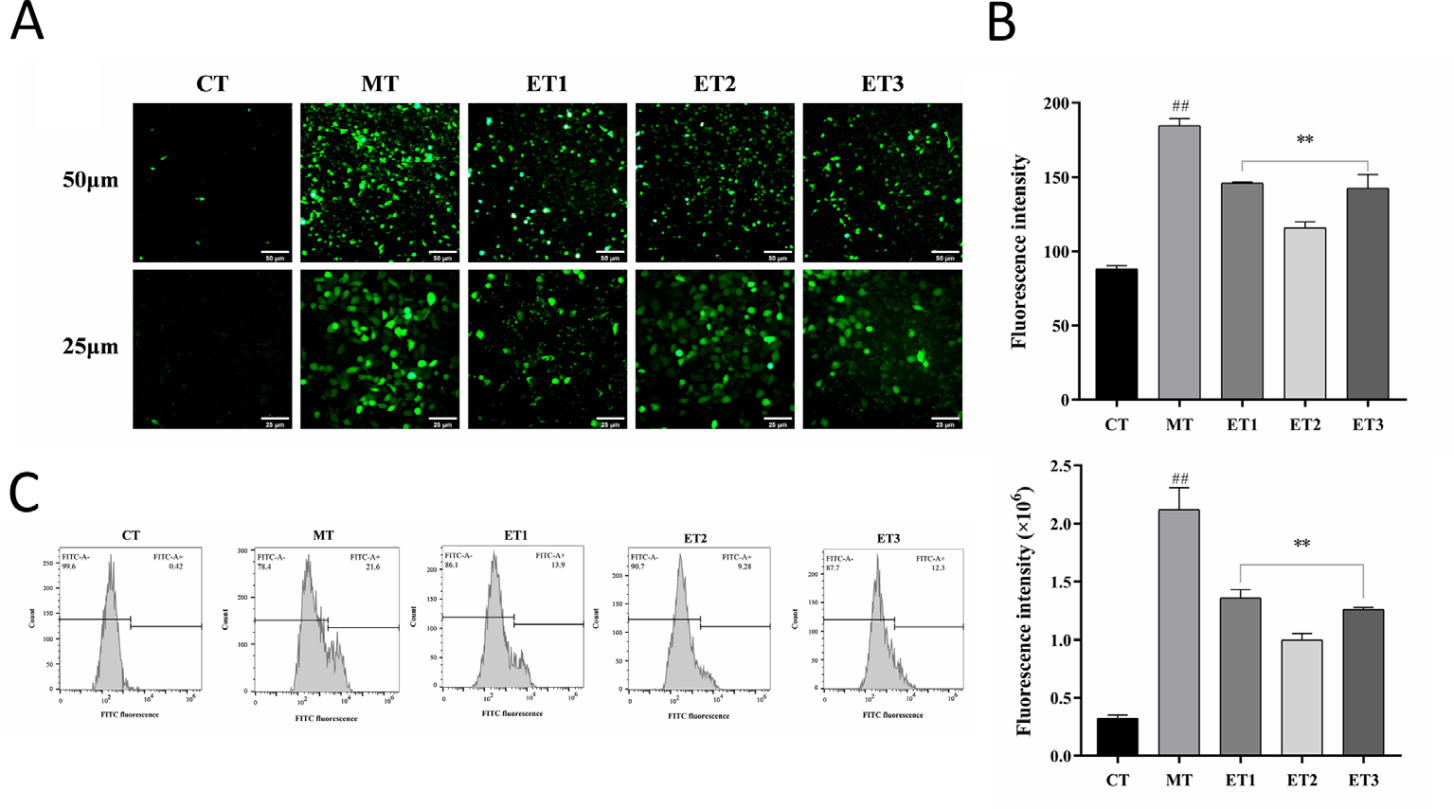
Effects of GF-CDs on intracellular reactive oxygen species (ROS) levels in oxidative loss. **(A)** Fluorescence staining of ROS; **(B)** Average fluorescence density; **(C)** ROS level measured by flow cytometry. All values were presented as means ± SD, and the significance of the difference were represented by asterisks and pound signs. CT, control group; MT, model group; ET1, experimental group 1; ET2, experimental group 2; ET3, experimental group 3. Compared with the control group, ^##^ *P*<0.01; Compared with the model group, **P<0.01.

The CFH-DA probe penetrates the cells, hydrolyzes by esterase, and oxidizes ROS to generate fluorescent substances. The ROS level in cells can be directly assessed by measuring the fluorescence intensity using a fluorescence microscope (Yu et al., 2021). **Figure 4A** depicts the results of the cells in each group, and **Figure 4B** illustrates the analysis results of average fluorescence density. The control group exhibited minimal fluorescence in the cells, while the model group displayed a substantial area of intense green fluorescence with discernible cell shape. The fluorescence regions of the three experimental groups were significantly smaller than those of the model group and exhibited weaker fluorescence intensity. The average fluorescence density of experimental group 2 was the lowest, with the least intracellular ROS content.

**Figure 4C** depicts the flow cytometry results. Compared with the control group, the peak diagram of the model group shifted rightward along the horizontal axis, and the fluorescence intensity exhibited significantly increased (*P*<0.01), indicating an increase in the ROS level. Compared with the model group, the peak plots of the three experimental groups shifted to the left of the horizontal axis, the fluorescence peak area was comparatively reduced, and the average fluorescence intensity was significantly weakened (*P*<0.01), indicating a decrease in ROS content. Experimental group 2 exhibited the weakest average fluorescence intensity among the three groups, consistent with the fluorescence staining results.

The results indicated that the concentration of GF-CDs in the experimental group reduced the ROS levels in oxidation-damaged cells, with the most pronounced effect observed at a concentration of 100 μg/mL.

### 3.5 Effects of GF-CDs on antioxidant indexes of oxidation-damaged cells

**Figure 5:**
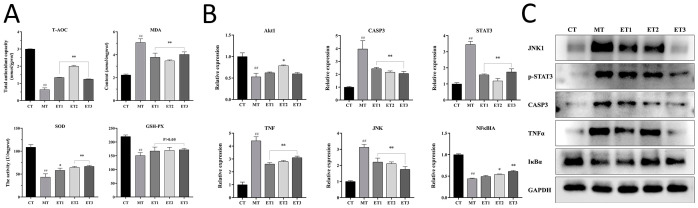
Effect of GF-CDs on antioxidant-related indexes and the expression of antioxidant-related genes and proteins. **(A)** Indexes related to antioxidation; **(B)** Expression of antioxidant-related genes; **(C)** Bands of antioxidant-related proteins in each group of cells. All values were presented as means ± SD, and the significance of the difference were represented by asterisks and pound signs. CT, control group; MT, model group; ET1, experimental group 1; ET2, experimental group 2; ET3, experimental group 3; T-AOC, Total antioxidant capacity; MDA, malonaldehyde; SOD, Superoxide Dismutase; GSH-Px, Gluta-thione peroxidase; TNF, tumor necrosis factor; Akt1, Serine/threonine kinase-α; STAT3, transcription 3; CAPS3, Caspase-3; JNK, c-Jun Nterminal kinase; NF-κBIA, the nuclear transcription factor-kappa-B; GAPDH, glycer-aldehyde-3-phosphate dehydrogenase. Compared with the control group, ^##^ *P* <0.01; Compared with the model group, *P <0.05, **P <0.01.

**Figure 5A** depicts the antioxidant-related indexes of cells in each group. The T-AOC level in the model group was significantly lower than that in the control group (*P*<0.01). The experimental group exhibited a significant increase in T-AOC level compared with the model group (*P*<0.01), and the highest level of T-AOC was 1.99 mM in experimental group 2.

The content of MDA was significantly increased in the model group than in the control group (*P*<0.01), and the degree of lipid peroxidation was higher. The content of MDA was significantly decreased in the experimental group than in the model group (*P*<0.01), and the degree of lipid peroxidation was alleviated.

The SOD activity was significantly lower in the model group than in the control group (*P*<0.01). The SOD activity was significantly increased in experimental group 1 than in the model group (*P*<0.05), and the difference between experimental groups 2 and 3 was significant (*P*<0.01), indicating that the GF-CDs improved the oxygen-free radical scavenging ability of injured cells.

Glutathione peroxidase (GSH-Px) vitality was significantly reduced in the model group than in the control group (*P*<0.01). No significant difference was observed in the activity of GSH-Px between the experimental group and the model group. However, both exhibited an increasing trend.

These results indicated that GF-CDs reduced MDA production in oxidation-damaged cells to a certain extent, increased SOD activity and T-AOC levels in cells, and played a significant role in antioxidant damage.

### 3.6 Effect of GF-CDs on the expression of antioxidant-related genes in oxidation-damaged cells

The expression levels of the six measured genes differed in various groups, and **Figure 5B** depicts the relative mRNA expression levels of these genes. The *serine/threonine kinase-α* (*AKT1*) gene expression level was significantly lower in the model group than in the control group (*P*<0.01), while the *AKT1* gene expression level was higher in experimental groups than in the model group. The experimental group 2 expression level was significantly higher than that in the model group (*P*<0.05). *Caspase-3* (*CASP3*) expression level, *signal transducer and transcription 3* (*STAT3*) activator, *tumor necrosis factor* (*TNF*), and *c-Jun N-terminal kinase* (*JNK*) genes were significantly higher in the model group than in the control group (*P*<0.01). The expression levels of these genes were significantly decreased in the three experimental groups than in the model group (*P*<0.01), and the decreasing trend of the JNK gene indicated a specific dose dependence. The expression of the inhibitory factor of the *nuclear transcription factor-kappa-B* (*NF-κBIA*) gene was significantly lower in the model group than in the control group (*P*<0.01). The expression level in the experimental groups 2 and 3 was significantly higher than that in the model group (*P*<0.05 and *P*<0.01, respectively).

### 3.7 Effect of GF-CDs on the expression of antioxidant-related proteins in oxidation-damaged cells

Western blot bands of antioxidation-related proteins in each group and GAPDH were used as the internal reference to obtain the relative expression levels of the proteins (**Figure 5C**). The results revealed that protein expressions of JNK1, phosphorylated STAT3 (P-STAT3) CASP3, and TNF-α were significantly increased in the model group than in the control group (*P*<0.01), while IκBα protein expression level was significantly lower in the model group than in the control group (*P*<0.05). JNK1 protein expression level was significantly decreased in the three experimental groups (*P*<0.01). The TNF-α protein expression level was significantly decreased in experimental group 1 than in the other groups (*P*<0.05). The CASP3 protein expression level was significantly decreased in experimental group 2 than in the other groups (*P*<0.01). CASP3 and TNF-α protein expression levels were significantly decreased in experimental group 3 than in the other groups (*P*<0.01). IκBα protein expression levels in the three experimental groups were not significantly different from those in the model group (*P*>0.05). However, the IκBα protein expression level was slightly higher in experimental groups 2 and 3 than in the model group.

### 3.8 Effect of GF-CDs on the apoptosis of oxidation-damaged cells

**Figure 6:**
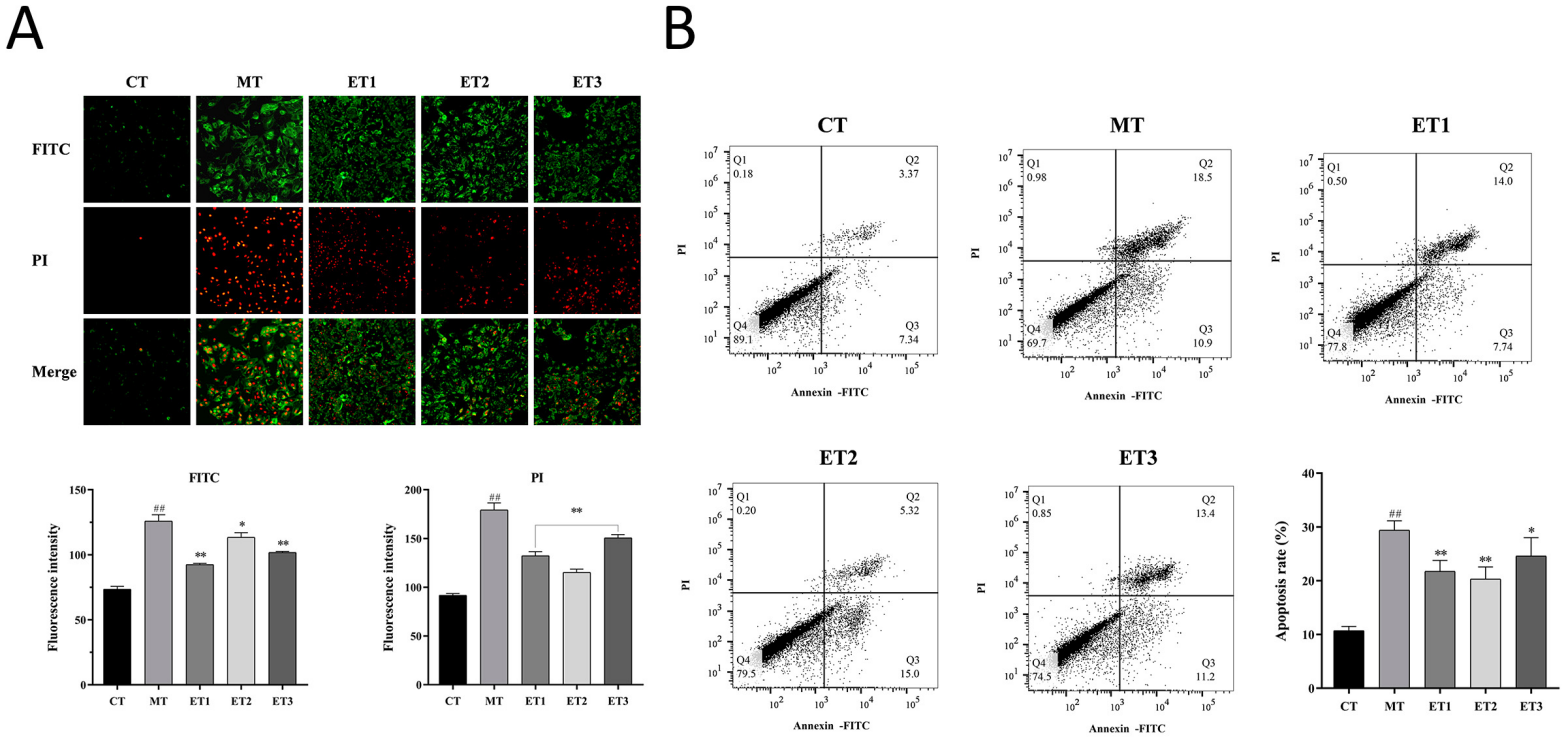
Effect of GF-CDs on apoptosis of cells with oxidative loss. **(A)** Apoptosis detection through fluorescence microscopy; **(B)** Detection of apoptosis by flow cytometry. All values were presented as means ± SD, and the significance of the difference were represented by asterisks and pound signs. CT, control group; MT, model group; ET1, experimental group 1; ET2, experimental group 2; ET3, experimental group 3; FITC, Annexin V-FITC; PI, Propidium Iodide. Compared with the control group, ^##^ *P* <0.01; Compared with the model group, *P <0.05, **P <0.01.

Cells stained with Annexin V-FITC alone were in the early stage of apoptosis and presented green fluorescence, whereas those without membrane integrity exhibited PI-stained nuclei and presented red fluorescence (**Figure 6A**). Cells in the late stage of apoptosis and necrosis were characterized by double positive staining through the combination of the two probes. The two fluorescence intensities presented in the model group were more significant than those in the control group, with increased apoptosis or necrosis. Double staining cells were significantly lower in the three experimental groups than in the model group, the intensity of green fluorescence was significantly decreased (*P*<0.05), and the apoptosis of the cells was alleviated.

The results of flow cytometry are depicted in (**Figure 6B**). Cells with single positive Annexin V-FITC (lower right Q3 quadrant) and double positive staining (upper right Q2 quadrant), namely apoptotic cells, were significantly more in the model group than in the control group. Apoptotic cells were decreased in the three experimental groups than in the model group, and the apoptotic rate was significantly reduced (*P*<0.05). The apoptotic rate in experimental group 2 was the lowest (20.32%).

The above results indicated that GF-CDs concentration in the experimental group inhibited cellular apoptosis under LPS stimulation and reduced the apoptosis rate.

## 4 Discussion

CDs have several characteristics, including excellent water solubility, low toxicity, excellent biocompatibility, and easy surface modification (Liu et al., 2020b; Sun et al., 2021; Li et al., 2022a). Its advantages include abundant raw materials, low synthetic costs, and environmentally sustainable practices. As the understanding of the properties of CDs deepens, research has increasingly focused on developing various CDs from natural sources and enhancing their optical properties. Studies utilizing traditional Chinese herbs through green synthesis to create CDs have emerged, which motivates our aim to prepare GF-CDs. A previous study reported that *Radix Sophorae* Flavescentis carbonate-based CDs can mitigate inflammation and oxidative stress while providing gastroprotective effects in rats with alcoholic exquisite acute gastric ulcer (Hu et al., 2021). Zhao et al. reported that *Paeoniae radix* Alba Carbonisata-derived CDs exhibited significant protective effects against acute liver injury induced by carbon tetrachloride (Zhao et al., 2020). In our previous study, a one-step hydrothermal method was used to synthesize the desired GF-CDs, and subsequent studies confirmed the role of GF-CDs in mitigating oxidative damage in IPEC-J2 cells, consistent with the results of other carbon dot studies.

The particle size of the GF-CDs generated in this experiment ranges from 2–7 nm, which meets the requirement for CDs dimensions (Quang et al., 2022). High-resolution transmission electron microscopy was used to examine the shape of GF-CDs, spheroid, and monodisperse in the solution, with good water solubility; the water solution is clear and light yellow. The absorption peaks of the CDs synthesized from green natural substances are primarily concentrated at 251–320 nm (Liu et al., 2019). The integrated spectrogram results indicate that GF-CDs exhibit minimal ultraviolet absorption and have abundant unsaturated double bonds (C=O bond, C=C bond), consistent with CDs synthesized in previous studies (Li et al., 2014; Yang et al., 2020; Liu et al., 2021). The surface functional groups of the prepared GF-CDs are abundant and may include hydroxyl, carbonyl, and aromatic structures. Previous studies have demonstrated that the surface groups of the synthesized CDs contain hydroxyl, carboxyl, carbonyl, and amino structures. CDs synthesized from natural sources predominantly consist of C, O, and N elements, with surface groups primarily being hydrophilic, which accounts for their excellent water solubility.

After adding GF-CDs solution to culture IPEC-J2 cells for 24 h, there was no significant change in cell viability (*P*>0.05), indicating that GF-CDs exhibited low toxicity, a characteristic of CDs. The results of other CDs studies are consistent with the results of this experiment. *Pueraria lobata* CDs (PLR-CDs) exhibited negligible toxicity to LO_2_ cells and RAW264.7 cells within a specific concentration range, demonstrating good biocompatibility (Wang et al., 2019). *Cirsium setosum* Carbonisata CDs (CSC-CDs) exhibited no significant toxic effect on RAW264.7 cells (Luo et al., 2018). CDs are expected to be developed and utilized in additional fields because of their low cytotoxicity and good biocompatibility.

ROS are the primary oxygen-free radical in living organisms, and the level can indicate the extent of oxidative damage in cells (Sul and Ra, 2021; Zhang et al., 2021; Kozlova et al., 2023). T-AOC represents the total capacity of various antioxidant macromolecules, small antioxidant molecules, and enzymes within living organisms (Mazdak et al., 2020; Liu et al., 2022). These antioxidants can eliminate ROS generated during physiological metabolism and impede the onset of ROS-generated oxidative stress (OS) (Li et al., 2022b). During OS in organisms, lipid peroxidation products decompose to form MDA (Guo et al., 2021; Shchulkin et al., 2022). Therefore, MDA is widely used as a biomarker for lipid oxidation detection. SOD and GSH-Px are members of the enzyme antioxidant system of the body that mitigates cell damage induced by excessive reactive oxygen-free radicals. SOD catalyzes superoxide radicals into molecular oxygen and hydrogen peroxide, while GSH-Px reduces lipid peroxides using reduced glutathione. Previous studies reported that SOD can inhibit the apoptosis of specific cells (Liu et al., 2020a; Miao et al., 2021; Wu et al., 2021). Various CDs were prepared from Chinese herbs, including *Radix sophorae* Flavescentis carbonisata-based CDs (Hu et al., 2021). *Paeoniae radix* Alba carbonisata-derived CDs (Zhao et al., 2020) and *Cirsium setosum* carbonisata CDs have significant antioxidant activities (Luo et al., 2018). Herein, GF-CDs exhibited similar antioxidant activity. GF-CDs inhibited the generation of ROS and MDA, increased T-AOC levels and the activities of SOD and GSH-Px, and significantly ameliorated cellular damage.

The signal transducer and activator of transcription 3 (STAT3) is the most pleiotropic member of the STAT protein family, which comprises the cytoplasmic transcription factors. The JAK/STAT signaling pathway associated with STAT3 can modulate cell cycle progression and proliferation (Kuo et al., 2015). AK2 phosphorylates LEPRb, STAT3, and other downstream molecules (Jiang et al., 2008). The downregulation of STAT3 enhances NOD, LRR, and pyrin domain-containing protein 3 (NLRP3)-mediated OS (Bai et al., 2018). The JNK is a subfamily of mitogen-activated protein kinases, including three gene subtypes (JNK1, JNK2, and JNK3). Multiple intracellular and extracellular stressors can activate JNK and directly phosphorylate downstream target proteins. JNK modulates cellular apoptosis, DNA repair, transcription, and various physiological processes (Kakemura and Igaki, 2024; Gazon et al., 2018). Cysteine protease 3 (Caspase 3) is a crucial member of the Caspase family and serves as the final executor of the apoptotic cascade. Their activation signifies the onset of apoptosis. Serine/threonine kinase-α (AKT1) is downstream of growth factors, oncogenes, and cellular stress responses, with its signaling pathway capable of regulating the cell cycle and apoptosis (Jeong et al., 2022). NF-κBIA is an essential factor that inhibits NF-κB activation, and its abnormal expression results in continuous NF-κB activation (Xu et al., 2024). Previous studies reported that upon activation, NF-κB translocates to the nucleus and binds to the enhancer or promoter regions of the target gene, thereby activating the expression of inflammatory response genes and genes associated with cellular stress, leading to the production of numerous inflammatory factors, including TNF-α and interleukin-1β. Besides, TNF-α and other cytokines can stimulate NF-κB-inducing kinase (NIK) and mitogen-activated protein kinase kinase-1 (MEKK1) to facilitate IκB phosphorylation, which is subsequently degraded by proteases, thereby liberating NF-κB for activation and further amplifying the signal (Li et al., 2022c). A previous study reported that TNF-α participates in the exogenous pathway of cell apoptosis and increases the ROS level, triggers an inflammatory response, and upregulates OS response (Wang et al., 2023). Numerous CDs, especially those derived from TCM, have exhibited pharmacological efficacy in anti-inflammatory actions or modulating inflammatory signaling pathways. *Radix sophorae Flavescentis carbonisata*-based CDs exhibited gastric protective effects by reducing NF-κB, TNF-α, IL-6, and inducible nitric oxide synthase (iNOS) levels to inhibit ethanol-induced inflammation and oxidative stress (Hu et al., 2021). Genistein-C-dots exhibited anti-inflammatory activity in LPS-stimulated macrophages, demonstrated by the suppression of pro-inflammatory cytokine levels and the upregulation of anti-inflammatory cytokine expression (Jaiswal et al., 2023). This study demonstrated that the mechanism underlying the antioxidative damage of GF-CDs may involve the inhibition of Caspase 3, STAT3, TNF-α, and JNK expression and promoting AKT1 expression.

CDs represent a heterogeneous category of nanomaterials with limitless production pathways. Before the formal application in biomedicine, substantial quantitative and standardized work remains to be completed, including the correlation between CDs precursors and their biological activity, the synthesis and purification methods employed, the physicochemical properties of the resulting CDs, and the effect of these parameters on the efficacy of the CDs across diverse applications. A typical example is that GF-CDs exhibit fluorescence properties. However, the fluorescence performance requires enhancement. Similar problems have been reported in other studies of CDs from natural sources. The fluorescence quantum yield can be enhanced by doping additional atoms or adding passivating agents to modify the surface of CDs (Pal et al., 2018; Yi et al., 2021). This study demonstrated that the fluorescence intensity of GF-CDs is the strongest at the excitation wavelength of 370 nm, with an optimal emission wavelength of approximately 450 nm. The fluorescence emission spectrum of GF-CDs exhibits excitation wavelength dependence.

## 5 Conclusion

**Figure 7:**
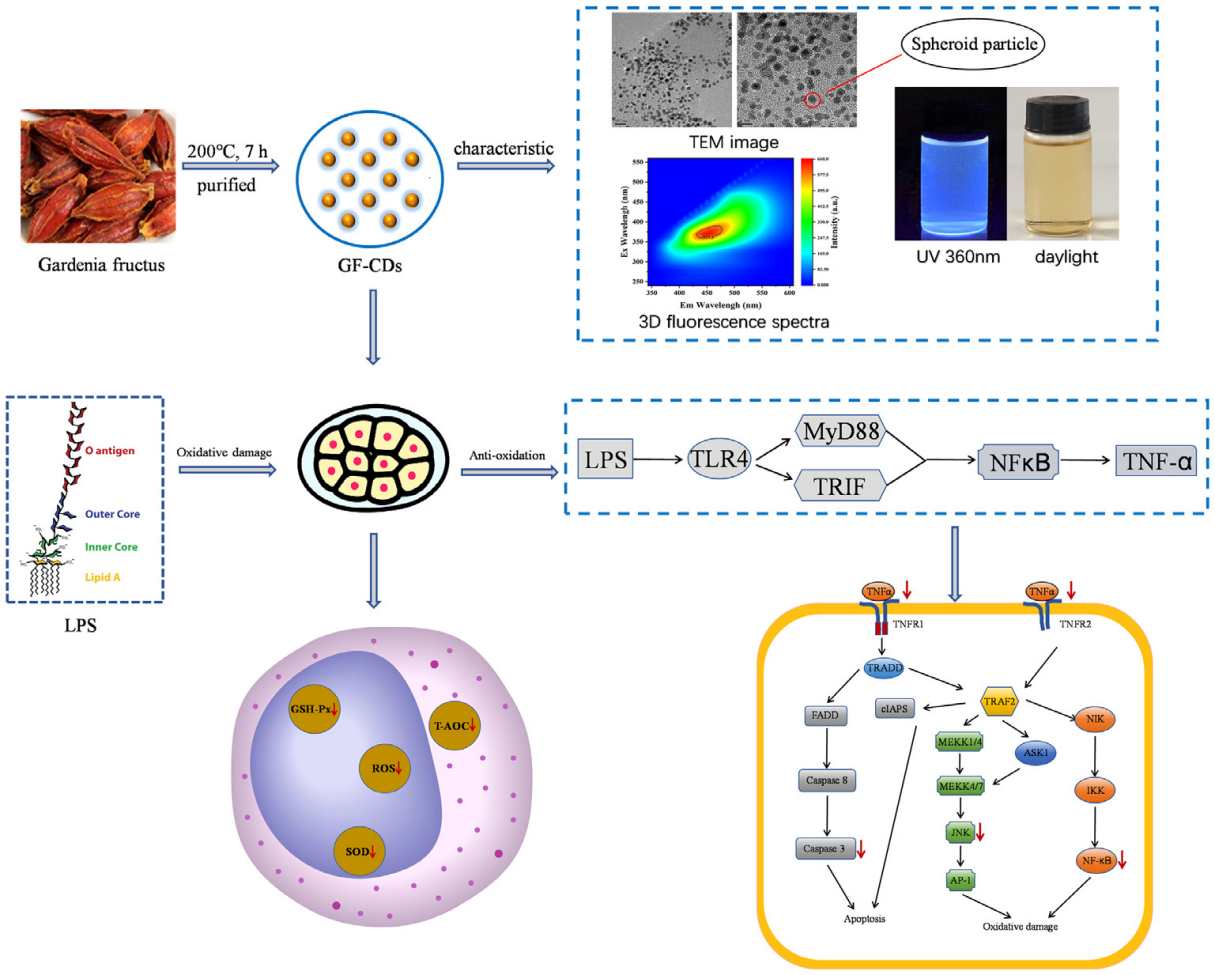
Possible mechanism of GF-CDs against oxidative stress-induced apoptosis in IPEC-J2 cells.

The GF-CDs was prepared by one-step hydrothermal method. The particle size of the GF-CDs was less than 7 nm, which had typical fluorescence characteristics, low toxicity and abundant hydrophilic groups on the surface. The GF-CDs has anti-apoptosis and anti-oxidation effects, and its mechanism is mainly related to up-regulation of AKT1 expression and down-regulation of Caspase 3, STAT3, TNF-α and JNK expression (**Figure 7**).

## Data Availability

The original contributions presented in the study are included in the article/supplementary material. Further inquiries can be directed to the corresponding author.
